# The BDNF Val66Met Polymorphism Does Not Increase Susceptibility to Activity-Based Anorexia in Rats

**DOI:** 10.3390/biology11050623

**Published:** 2022-04-19

**Authors:** Carla L. Pietrucci, Laura K. Milton, Erika Greaves, Aneta Stefanidis, Maarten van den Buuse, Brian J. Oldfield, Claire J. Foldi

**Affiliations:** 1 Department of Physiology, Monash University, Clayton 3800, Australia; carlalouisepietrucci@gmail.com (C.L.P.); laura.milton@monash.edu (L.K.M.); erika.greaves@monash.edu (E.G.); aneta.stefanidis@monash.edu (A.S.); brian.oldfield@monash.edu (B.J.O.); 2 Metabolism, Diabetes and Obesity Program, Monash Biomedicine Discovery Institute, Clayton 3800, Australia; 3 School of Psychology and Public Health, La Trobe University, Kingsbury Drive, Bundoora 3086, Australia; m.vandenbuuse@latrobe.edu.au

**Keywords:** anorexia nervosa, activity-based anorexia, brain-derived neurotrophic factor, 66Met, animal models, feeding

## Abstract

**Simple Summary:**

Genetic animal models are a valuable tool for understanding how human pathologies develop. The type of animal model chosen is important for uncovering effects specific to certain behaviours and neurobiological functions. A polymorphism in the brain-derived neurotrophic factor (BDNF) has been linked with various clinical conditions in human subjects and with mouse models of anorectic behaviour. This study investigated for the first time the role of the BDNF Val66Met allelic substitution in a rat model of anorexia nervosa (AN), known as activity-based anorexia (ABA). Contrary to reports of altered BDNF signaling in patients with AN and increased anorectic behaviour in a mouse model containing the same allelic variation, it showed that 66Met did not alter susceptibility to weight loss or aspects of energy balance, including feeding and exercise in the rat model. It highlights the need to consider species–specific differences when evaluating animal models of human pathologies.

**Abstract:**

Brain-derived neurotrophic factor (BDNF) is abundantly expressed in brain regions involved in both homeostatic and hedonic feeding, and it circulates at reduced levels in patients with anorexia nervosa (AN). A single nucleotide polymorphism in the gene encoding for BDNF (Val66Met) has been associated with worse outcomes in patients with AN, and it is shown to promote anorectic behaviour in a mouse model of caloric restriction paired with social isolation stress. Previous animal models of the Val66Met polymorphism have been in mice because of the greater ease in modification of the mouse genome, however, the most widely-accepted animal model of AN, known as activity-based anorexia (ABA), is most commonly conducted in rats. Here, we examine ABA outcomes in a novel rat model of the BDNF Val66Met allelic variation (Val68Met), and we investigate the role of this polymorphism in feeding, food choice and sucrose preference, and energy expenditure. We demonstrate that the BDNF Val68Met polymorphism does not influence susceptibility to ABA or any aspect of feeding behaviour. The discrepancy between these results and previous reports in mice may relate to species–specific differences in stress reactivity.

## 1. Introduction

Anorexia nervosa (AN) has among the highest mortality rates of psychiatric disorders [1], and it is characterized by extreme caloric restriction and compulsive exercise behaviour that results in dangerously low levels of body weight [2]. This relentless pursuit of weight loss is exacerbated by an overwhelming fear of weight gain and an inability of patients to recognise the severity of their own body weight condition [3,4]. Although AN was historically considered a purely psychosocial issue, it is now clear that there are strong neurobiological and genetic drivers. For example, functional imaging studies in AN patients demonstrate reduced activity in ventral striatal reward regions [5,6] and excessive activity in prefrontal control regions [7,8]. Additionally, AN is highly heritable [9,10], the proportion of which has been estimated at 58% [11], however, this likely varies within the range of 28–74% [12]. Recent genome wide association studies (GWAS) have uncovered the first significant risk genes associated with AN [13,14], including genes with known functions in feeding behaviour and reward [15]. These studies provide robust evidence for a genetic aetiology of AN, and they show strong genetic correlations with other psychiatric conditions including schizophrenia and obsessive–compulsive disorder as well as with metabolic traits, including leptin and insulin regulation and type-2 diabetes. This has led to the reconceptualization of AN as a “metabo-psychiatric illness” and signposts the importance of investigating factors that influence both psychiatric and metabolic phenotypes involved in the development of AN.

Brain derived neurotrophic factor (BDNF) is involved in several metabolic and psychiatric functions, and it plays multiple roles in the regulation of homeostatic and reward-related feeding behaviour, providing a rationale to investigate its influence in the aetiology of AN. Predominantly responsible for neuron growth, function and survival, BDNF is the most abundant growth factor in the central nervous system (CNS), and it specifically acts to stimulate neurogenesis and synaptic plasticity in the brain [16,17]. Differences in the expression and function of BDNF have been found to alter the regulation of food intake and of body weight [18], and to drive the development of many psychiatric conditions, including schizophrenia, post-traumatic stress disorder, and mood disorders [19,20,21,22]. Decreased serum BDNF has been consistently reported in AN patients compared to controls [23,24], although this difference is ameliorated with body weight recovery [25], suggesting disrupted BDNF signalling is associated with the acute phase of the illness [26]. Highly expressed within specific nuclei of the hypothalamus [27,28,29], BDNF binds to the TrkB receptor and stimulates proopiomelanocortin (POMC) neuronal activity in order to decrease food intake and to increase energy expenditure [27,28,29,30]. It is also abundantly expressed in dopamine neurons in the ventral tegmental area (VTA), and it regulates hedonic feeding [28,31]. The actions of BDNF in both the hypothalamic and the midbrain reward circuits suggest it is a plausible contributor to the pathophysiology of AN, considering that AN is associated with deficits in both homeostatic and reward-based feeding.

However, there seems to be a discrepancy between peripheral BDNF levels in patients with AN and the known actions of BDNF within the hypothalamus and the reward neurocircuitry on feeding behaviour and appetite. As mentioned above, serum BDNF levels are significantly lower in AN patients compared to healthy controls [32]. This raises the question of how accurately circulating BDNF levels reflect BDNF signalling in the brain, considering that reduced BDNF signalling in the hypothalamus stimulates food intake, and selective deletion of BDNF in the VTA triggers increased hedonic feeding, both of which promote weight gain and obesity [28,33]. These variations between central and peripheral BDNF levels may be associated with the activity-dependent release of BDNF [19,33,34] and one modifier of release is the Val66Met single nucleotide polymorphism (SNP). This SNP within the gene encoding for BDNF has been previously associated with other psychiatric disorders such as schizophrenia, anxiety, and depression [35,36,37,38]; and, it has been linked with impaired neurocognitive function in healthy adults [39]. The SNP occurs in the proBDNF protein, at position rs6265, causing the replacement of Valine (Val) with Methionine (Met) at codon position 66 (Val66Met) within exon 5 of the human BDNF gene [40,41,42]. Preliminary support for the involvement of the BDNF Val66Met polymorphism in AN was identified in 2003 [42] and, although the Met allele is not more common in female AN patients compared to controls, AN Met carriers exhibit more severe disorder-specific symptoms [3]. Other brain functional outcomes relevant to AN have also been shown in females carrying 66Met compared to Val allele controls, including alterations in dopaminergic reward processing and stress responsivity [43]. While this evidence supports a role for BDNF Val66Met in AN, animal models are required to systematically interrogate the effects of this risk variant on anorectic behaviours.

The activity-based anorexia (ABA) model is the most well established and widely used animal model of AN, and it is characterized by high levels of physical activity despite minimal food intake, which results in dramatic weight loss [4,44,45,46,47,48]. A typical ABA paradigm includes unlimited running wheel access combined with time-limited access to food (usually 90 min per day). It is important to note that either running wheel access or time-limited access to food alone does not produce the ABA phenotype; but, hyperactivity occurs at the onset of time-restricted feeding, precipitating a lack of control over energy balance that without intervention leads to death [4,44,45,46,47,48]. Central BDNF expression in the VTA is elevated in mice in response to running wheel access and decreased in the prefrontal cortex in response to food restriction, however, no regionally-specific changes in BDNF have been associated with the combination of wheel access and food restriction that defines the ABA paradigm [49]. Studies on the role of the BDNF Val66Met polymorphism in mouse models of AN have shown inconsistent results, with one study showing the Met allele to be associated with reduced vulnerability to ABA in male mice [44]. In contrast, a model of 20–30% caloric restriction showed increased anorectic behaviours in female Met carriers but only when combined with adolescent social isolation stress [47].

Recently, a novel rat model of the Val66Met polymorphism has been developed carrying a valine to methionine substitution in the rat BDNF gene (Val68Met) [50]. Because rats have two additional threonine amino acids at positions 57 and 58, this rat Val68Met is the physiologically relevant equivalent to the human Val66Met SNP. A recent comprehensive study revealed that adult BDNF rats with the Met/Met genotype have a specific impairment in conditioned fear memory compared to Val/Met heterozygotes and Val/Val controls that does not extend to differences in fear learning, extinction, or anxiety-like behaviour [51]. In the present study, we aimed to characterise the effects of the Val68Met rat polymorphism on susceptibility to pathological weight loss in ABA and determine its effects on homeostatic and hedonic feeding, running wheel activity, and markers of energy expenditure in brown adipose tissue (BAT). The ABA model offers advantages over the previously described mouse model of AN because it does not rely on imposed food restriction but rather the tendency of animals exposed to the paradigm to choose to run instead of eat. We conclude that the BDNF gene variant does not increase susceptibility to ABA or cause a change in food intake or preference in female rats, nor does it elicit a change in expression of uncoupling protein 1 (UCP1) in BAT, a marker of thermogenic activity. These results contrast with previous reports in mouse models and we propose that the discrepancy may be linked to between-species differences in stress vulnerability.

## 2. Materials and Methods

### 2.1. Animals and Housing

The BDNF Val68Met rat was generated on a Sprague Dawley background through nuclease-mediated genome editing technology (Cyagen; Santa Clara, CA, USA), creating an amino acid substitution of Valine in place of Methionine at codon position 68 of the BDNF pro-form. Adult heterozygous BDNF Val68Met rats (*n* = 6 male, *n* = 6 female) were obtained from the ARC (Animal Resources Centre, WA, Australia), and they were used to produce offspring of all three genotypes. Genotyping of tail tips from offspring at postnatal day 10 was outsourced to Transnetyx (Cordova, TN, USA). Female offspring (*n* = 44, from 8 separate litters) of all 3 genotypes [Val/Val (wildtype; *n* = 12), Val/Met (heterozygous substitution; *n* = 16) or Met/Met (homozygous substitution; *n* = 16)] underwent behavioural testing between 5–16 weeks of age. Rats were housed in groups until behavioural testing in opaque polypropylene cages (32 × 32 × 40 cm) in a humidity (60 ± 10%) and temperature (21.5–23.5 °C) controlled room under a 12-h light/dark cycle (lights off at 1300 h). Rats were subsequently individually housed in testing chambers as described below. All animals had ad libitum access to food (standard laboratory rodent chow; Barastoc Feeds, Melbourne, VIC, Australia) and water unless otherwise specified. All experimental procedures complied with the Australian code for the care and use of animals for scientific purposes and were approved by the Monash Animal Resource Platform Ethics Committee (MARP 2018-026). Animals and/or data points were excluded from analyses for the following reasons: during exposure to ABA conditions rats gained body weight (*n* = 2) or running wheel activity was not recorded (*n* = 1), and during feeding and preference studies food hoarding behaviour was noted (Baseline; *n* = 4, high fat diet preference; *n* = 5, sucrose preference; *n* = 1). 

### 2.2. Activity-Based Anorexia (ABA)

At 5 weeks of age, female (*n* = 22) BDNF Val68Met rats were housed individually in standard opaque polypropylene cages (32 × 32 × 40 cm) for 7 days before being transferred to transparent activity wheel living chambers (Lafayette Instruments, model No. 80859) and ad libitum food access for 6 days to acclimate to the running wheel. Running wheel activity (RWA) in cage mounted wheels was recorded with Activity Wheel Software (Lafayette Instruments, Lafayette, IN, USA) at 10 min intervals for the duration of the experiment. At 7 weeks of age, food access was restricted to 90 min/day at the onset of the dark cycle (1500–1630 h) to initiate activity-based anorexia (ABA). Rats were allowed to lose a total of 20–25% of baseline body weight before full food access was restored. When rats had returned to ≥100% baseline body weight they were euthanized with sodium pentobarbitone (Lethabarb 300 mg/kg; Virbac, Milperra, NSW, Australia) and BAT samples collected.

### 2.3. Feeding Patterns and Behaviour

At 10 weeks of age, a separate cohort of female (*n* = 22) BDNF Val68Met rats were individually housed in BioDAQ automated feeding cages (Research Diets, Inc., New Brunswick, NJ, USA) with two adjacent food or liquid hoppers that allowed continuous recording of consummatory behaviour. Baseline feeding patterns were recorded over a 7-day period with ad libitum access to food and water. Subsequently, rats were subject to palatable food and sucrose preference tests for 24 h, each preceded by a 24 h control side preference test. For side preference examination, both adjacent hoppers were filled with either standard chow or tap water; and, for choice tests, a single hopper was replaced with one containing high-fat food (SF04-001; Specialty Feeds, Glen Forrest, WA, Australia) or 1.5% sucrose solution on the non-preferred side (if there was evidence of a side preference). Following testing, rats were euthanized with sodium pentobarbitone and BAT samples collected as above.

### 2.4. Measurements of UCP1 Protein in Adipose Tissue

Interscapular BAT pads (*n* = 29) were removed from a subset of animals following completion of behavioural testing and immediately frozen on dry ice. As previously described, protein was extracted [52] by homogenization of BAT samples (100 mg) in RIPA lysis and an extraction buffer (product 89990, Thermo Fisher Scientific, Scoresby, VIC, Australia) that contained protease (1:50 complete tablet; Roche, Basel, Switzerland), and the supernatant containing cytosolic proteins was carefully removed. Protein concentrations were determined using the Bicinchoninic Acid (BCA) Protein Assay Kit (Thermo Fisher Scientific, Waltham, MA, USA) and proteins were solubilised in 4× Laemlli’s buffer. Protein (20 μg) was loaded onto a Mini-PROTEAN TGX Stain-Free gel (Bio-Rad Laboratories, Hercules, CA, USA) and transferred onto a PVDF membrane (Bio-Rad Laboratories, Hercules, CA, USA). The membrane was blocked in 1% skim milk powder in Tris-buffered saline/1% Tween 20 (TBST) and incubated overnight at 4 °C with primary antisera raised against UCP1 (ab10983 Rb; Abcam, Cambridge, UK; diluted 1:1000) in 1% bovine serum albumin (BSA) and TBST. Following secondary antibody incubation using antirabbit horseradish peroxidase (HRP) (NA9340V; Abcam, Waltham, MA, USA; 1:4000) in 1% BSA and TBST, protein expression signals were visualised on an enhanced chemiluminescence (ECL) substrate using the ChemiDoc XRS Imaging system (Bio-Rad Laboratories, Hercules, CA, USA). Immunoreactive UCP1 protein expression was quantified from membrane images where all replicates were normalised against their respective total protein content (ChemiDoc MP and ImageLab software version 6.1, Bio-Rad Laboratories, Hercules, CA, USA).

### 2.5. Statistical Analyses

#### 2.5.1. Activity-Based Anorexia

Survival was evaluated with a log-rank (Mantel-Cox) χ^2^ test. Analysis of variance (ANOVA) was performed to analyse the effects of BDNF Val68Met on body weight and food intake changes over baseline and ABA experimental phases. One-way repeated measures ANOVA followed by post hoc multiple comparisons with a Tukey’s correction were used to analyse body weight loss, food intake, and daily running wheel measures between experimental phases. Two-way repeated measures ANOVA followed by post hoc multiple comparisons with a Tukey’s correction were used to analyse hourly RWA. Two-way repeated measures ANOVA followed by post hoc multiple comparisons with a Sidak’s correction were used to analyse daily RWA and food anticipatory activity (FAA). The significance for all tests was set at *p* < 0.05.

#### 2.5.2. Feeding Patterns and Behaviour

One-way repeated measures ANOVA followed by post hoc multiple comparisons with a Tukey’s correction were used to analyse the effects of BDNF Val68Met on food and liquid intake. Two-way repeated measures ANOVA followed by post hoc multiple comparisons with a Tukey’s correction were used to analyse body weight gain. Two-way repeated measures ANOVA followed by post hoc multiple comparisons with a Bonferroni’s correction were used to analyse the proportion of chow vs. HFD intake and water vs. sucrose intake. The significance for all tests was set at *p* < 0.05.

#### 2.5.3. Western Blots

One-way repeated measures ANOVA followed by post hoc multiple comparisons with a Tukey’s correction were used to analyse the effects of BDNF Val68Met on UCP1 expression in BAT samples.

## 3. Results

### 3.1. Effects of BDNF Val68Met on ABA Outcomes

**Figure 1:**
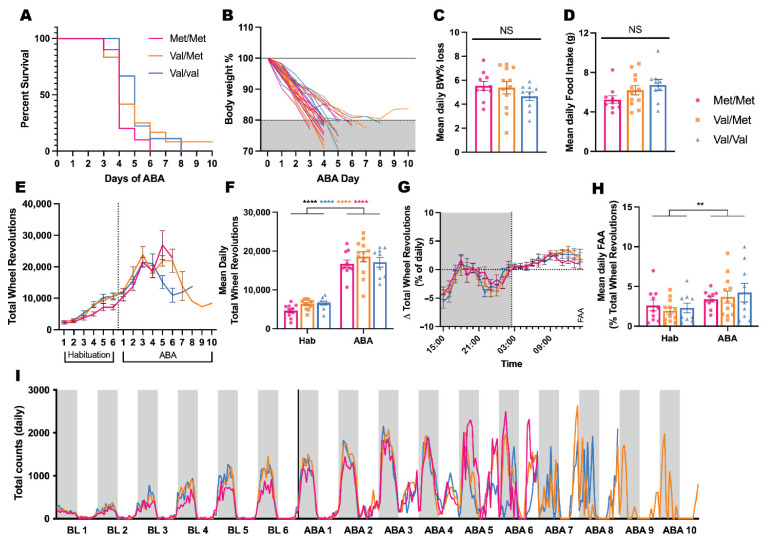
Possession of the BDNF Met allele does not influence susceptibility to activity-based anorexia in female rats. (**A**) Survival in ABA was not significantly different between genotypes; Log-rank (Mantel-Cox) χ^2^(2) = 2.482, *p* = 0.2892. (**B**) Body weight as a percentage of baseline for all individual animals. Neither mean daily body weight % loss (**C**) nor food intake (**D**) were significantly different between genotypes; One-Way ANOVA; (**C**) *F*(2, 28) = 0.9957, *p* = 0.3822; (**D**) *F*(2, 28) = 2.253, *p* = 0.1239. (**E**) Group mean ± SEM of daily RWA (total wheel revolutions). (**F**) Mean daily RWA during Habituation and ABA; Two-Way ANOVA, Phase *F*(1, 28) = 321.4, *p* < 0.0001 (Bonferroni’s multiple comparisons, all genotypes *p* < 0.0001), Genotype *F*(2, 28) = 1.455, *p* = 0.2504, Interaction *F*(2, 28) = 0.6503, *p* = 0.5296. (**G**) Group mean ± SEM of change in mean hourly RWA as a proportion of total daily RWA from Habituation to ABA. (**H**) Mean daily FAA during Habituation and ABA; Two-Way ANOVA, Phase *F*(1, 28) = 8.799, *p* = 0.0061, Genotype *F*(2, 28) = 0.1885, *p* = 0.8292, Interaction *F*(2, 28) = 0.4710, *p* = 0.6292. (**I**) Group mean of mean hourly RWA across the entirety of the experimental period. ** *p* < 0.01, **** *p* < 0.0001, NS not significant *p* > 0.05.

Exposure to ABA conditions produced rapid weight loss and hyperactivity, consistent with the primary features of the model. There was no significant effect of possession of either one or both Met alleles on ABA outcome measures, including survival (Figure 1A; *p* = 0.2892), body weight loss (Figure 1B,C; *p* = 0.3822;), food intake (Figure 1D; *p* = 0.1239), or running activity (Figure 1E–G; *p* = 0.2504), indicating that the BDNF Val68Met polymorphism had no influence over susceptibility to pathological weight loss. While all animals increased running activity dramatically at the onset of food restriction in ABA (Figure 1E,F; all *p*s < 0.0001), the extent and the timing (Figure 1G–I) of starvation-induced hyperactivity, including the development of FAA (Figure 1H; *p* = 0.8292), was not influenced by genotype.

### 3.2. Effects of BDNF Val68Met on Feeding Patterns and Behaviour

**Figure 2:**
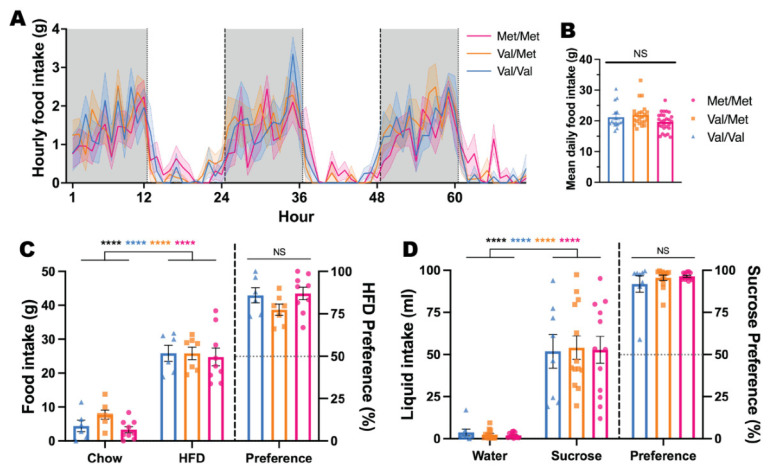
Possession of the BDNF Met allele does not alter homeostatic feeding or preference for highly palatable food/solution. (**A**) Group mean ± SEM of mean hourly food intake across 3 consecutive days; Two-Way ANOVA, Time *F*(12.14, 242.7) = 11.46, *p* < 0.0001, Genotype *F*(2, 20) = 1.318, *p* = 0.2900, Interaction *F*(140, 1400) = 0.8682, *p* = 0.8580. (**B**) Mean daily food intake was not significantly different between genotypes; One-Way ANOVA *F*(2, 66) = 2.932, *p* = 0.0603. Food (**C**) and Liquid (**D**) intake and preference during independent 24-h preference tests. Two-Way ANOVA performed on intake data; One-Way ANOVA performed on preference data. C) Intake: Food *F*(1, 19) = 134.4, *p* < 0.0001 (Bonferroni’s multiple comparisons, all genotypes *p* < 0.0001), Genotype *F*(2, 19) = 1.442, *p* = 0.2613, Interaction *F*(2, 19) = 0.4114, *p* = 0.6685. Preference: *F*(2, 19) = 1.890, *p* = 0.1783. (**D**) Intake: Liquid *F*(1, 30) = 109.5, *p* < 0.0001 (Bonferroni’s multiple comparisons, all genotypes *p* < 0.0001), Genotype *F*(2, 30) = 0.01264, *p* = 0.9874, Interaction *F*(2, 30) = 0.04170, *p* = 0.9592. Preference: *F*(2, 30) = 0.9142, *p* = 0.4117. **** *p* < 0.0001, NS not significant *p* > 0.05.

Food intake in this study was largely restricted to the dark phase, consistent with normal feeding patterns in laboratory rodents, and all animals showed strong preferences for high-fat food over regular chow and for sucrose-sweetened water over regular water. There were no effects of the BDNF Val68Met polymorphism on homeostatic or reward-related feeding behaviour, with similar intake of food at baseline for all genotypes across the three-day measurement period (*p* = 0.2900; Figure 2A) and on average (*p* = 0.0603; Figure 2B). All animals, regardless of genotype, had significantly greater intake of HFD over chow (*p* < 0.0001; Figure 2C) and sucrose solution intake over regular water (*p* < 0.0001; Figure 2D), however, there were no significant differences between preference scores between genotype groups (both *p*s > 0.1783).

### 3.3. Effects of BDNF Val68Met on UCP1 Expression in BAT

**Figure 3:**
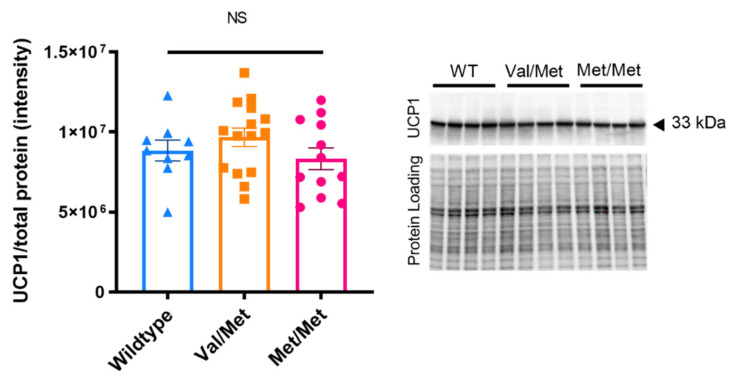
Possession of the BDNF Met allele does not alter expression of UCP1 in BAT. Group mean ± SEM of UCP1 expression expressed as a proportion of total protein; One-way ANOVA *F*(2, 33) = 1.253, *p* = 0.298. Blot shows example of staining for protein (one of three gels analysed). Both original images are available to view in Supplementary Materials (Figures S1 and S2).

The expression of UCP1 in BAT tissue was unchanged by allelic variation of the BDNF gene, with a similar expression to wildtypes seen in heterozygote and homozygote Met carriers (Figure 3; *p* = 0.298).

## 4. Discussion

The major findings of this study demonstrate that the BDNF Val68Met polymorphism had no effect on the susceptibility of adolescent female rats to body weight loss in the ABA paradigm. Body weight loss was similar for all genotype groups following commencement of ABA feeding restrictions. The BDNF polymorphism had minimal effects on normal feeding behaviour, and did not induce a change in hedonic feeding, in as much as preference for high fat food and sucrose-sweetened water was high for all animals regardless of genotype. Additionally, no phenotypic differences were exhibited between homozygote and heterozygote Met allele carriers throughout all experimental tests, suggesting there is no “dosing” effect of having both allelic substitutions. This is in keeping with the evidence from previous mouse studies that revealed no differences in anorectic behaviour between heterozygotes and homozygote Met carriers [47]. Moreover, these results demonstrate that the thermogenic capacity of interscapular BAT was unchanged by 66Met; however, based on this study it may be premature to conclude that energy expenditure and metabolic function are not influenced by possession of this allelic substitution. It is possible that changes in muscle and/or white adipose tissue (WAT) function contribute to metabolic outcomes of 66Met in ABA rats, considering that physical exercise alters WAT morphology in BDNF Met/Met mice [53] and that exposure to ABA conditions has been shown to increase UCP1 expression in WAT depots [54]. Taken together, these outcomes indicate that the BDNF Val68Met polymorphism in rats does not influence feeding or body weight under normal conditions or when exposed to the ABA paradigm, and suggest that other tissue types could be examined for analysis of energy expenditure in future studies. This contrasts with the effects of the BDNF Val66Met polymorphism on anorectic behaviour in mice [44,47], and it highlights a number of methodological considerations for further investigation.

In designing these experiments, we recapitulated the Madra & Zeltser [47] protocol that involved social isolation stress at 5 weeks of age prior to caloric restriction at 7 weeks of age, in which mice received 20–30% less chow than their standard daily intake for 10 days. The findings of this study indicated that aphagic episodes (AE; periods of complete refusal to eat) occurred only in mice that had been socially isolated from 5 weeks of age and that Met carriers had three times more AE compared to wildtype mice, indicating a combinatorial effect of the BDNF polymorphism with stress and caloric restriction. In other words, a gene–environment interaction was critical in mediating the effects of caloric restriction on anorectic behaviour in BDNF 66Met mice. In our attempt to mimic this protocol in combination with the ABA paradigm, we singly housed female BDNF Val68Met rats at 5 weeks of age before exposing them to ABA conditions, consisting of unlimited running wheel access with time-restricted food access for 90 min/day at 7 weeks of age. The absence of significant differences between genotypes on feeding behaviour and susceptibility to pathological weight loss in the present study may be related to species–specific differences in stress reactivity, considering that mice exhibit greater anxiety-like behaviours than rats when they are being handled, adapting to environmental changes, and when learning a new task [55]. Indeed, there is evidence to suggest that the period of adolescent development at which the ABA model is most effective (between 6–8 weeks of age) seems to be associated with a specific stress resilience in Sprague-Dawley rats [56]. Therefore, the effects of social isolation in the present study may not have been stressful enough for rats to elicit the same gene–environment interaction that was required to produce anorectic behaviour in BDNF 66Met mice. It may also be that access to a running wheel alleviated the stress of social isolation in the present study [57], considering that exercise is shown to decrease anxiety and depressive-like symptoms by reducing the hypothalamic–pituitary–adrenal (HPA) axis response to stress [58]. Moreover, while male BDNF Met/Met mice were shown to display increased anxiety-like behaviour prior to ABA exposure, this was ameliorated following ABA, and in fact male Met/Met mice displayed reduced vulnerability to body weight loss in ABA due to the absence of starvation-induced hyperactivity [44]. Thus, running wheel access may be protective against an anxiety phenotype generated by the BDNF allelic variation.

One consideration when investigating genetic variants that are likely to be sensitive to stress in rodent models is that experimenter intervention itself can have dramatic effects on behavioural and on physiological phenotypes and that this can vary between studies based on environmental conditions and idiosyncratic handling techniques. These influences are likely to contribute to the less than ideal levels of reproducibility within and between laboratories that have been a focus of recent attention in behavioural neuroscience. In agreement with this focus, we believe that considerable efforts should be made to automate as many procedures in behavioural testing protocols as possible to avoid these confounds. Based on the known interaction between stress and BDNF signalling [59], it is plausible that additional standardisation methods are required to elucidate a reliable phenotype in the BDNF Val68Met rat model and that effects of the BDNF polymorphism on susceptibility to ABA in rats may require a more profound or prolonged stress exposure.

## 5. Conclusions

The widespread use of genetically-modified mouse models has shown utility for understanding disease mechanisms and for identifying therapeutic targets for a range of human pathologies. However, the present study suggests a cautious approach should be taken when attempting to generalize phenotypes based on single nucleotide polymorphisms even between rodent species. This study aimed to improve on genetic modification strategies for understanding the role of BDNF allelic variation by using a model with a physiologically relevant Met substitution, rather than the human gene knockin mouse model. Moreover, we aimed to use the most ethologically relevant and widely accepted rodent model of AN, which elicits voluntary reductions in food intake rather than imposed food restriction. That no phenotype related to feeding behaviour or anorexia was uncovered in this study does not imply that the effects of BDNF Val68Met will not be revealed in future studies, under the right conditions and with even more appropriate tools. The ultimate goal is to utilise the power of animal models to gain a deeper understanding of the behavioural and the metabolic underpinnings of AN in order to inform the development of novel therapeutic strategies.

## Data Availability

All data supporting reported results can be provided on request. BDNF rs6265 Met/Met breeding pairs can be shared by request to Caryl E. Sortwell, Ph.D., Michigan State University, sortwell@msu.edu.

## Supplementary Materials

The following supporting information can be downloaded at: https://www.mdpi.com/article/10.3390/biology11050623/s1, Figures S1 and S2: Original images of Full Western Blot.
