# Supplementary figures and images for: The BDNF Val66Met Polymorphism Does Not Increase Susceptibility to Activity-Based Anorexia in Rats
Source: Biology (Basel). 2022 Apr 19;11(5):623. doi: 10.3390/biology11050623 (PMC9138045; doi:10.3390/biology11050623)

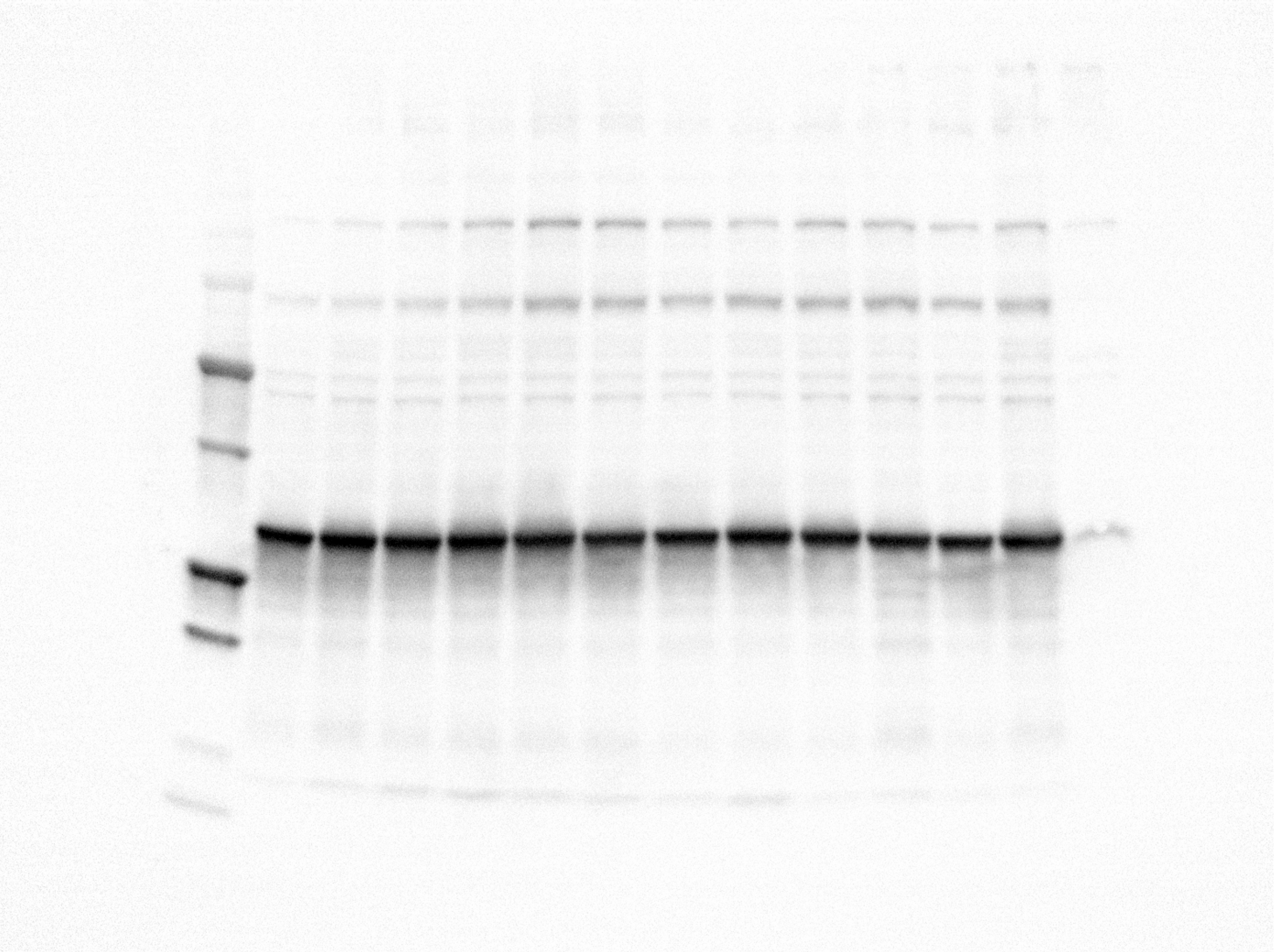

Supplement: Supplementary file 1 [file biology-11-00623-s001.zip › Figure S1.tif]

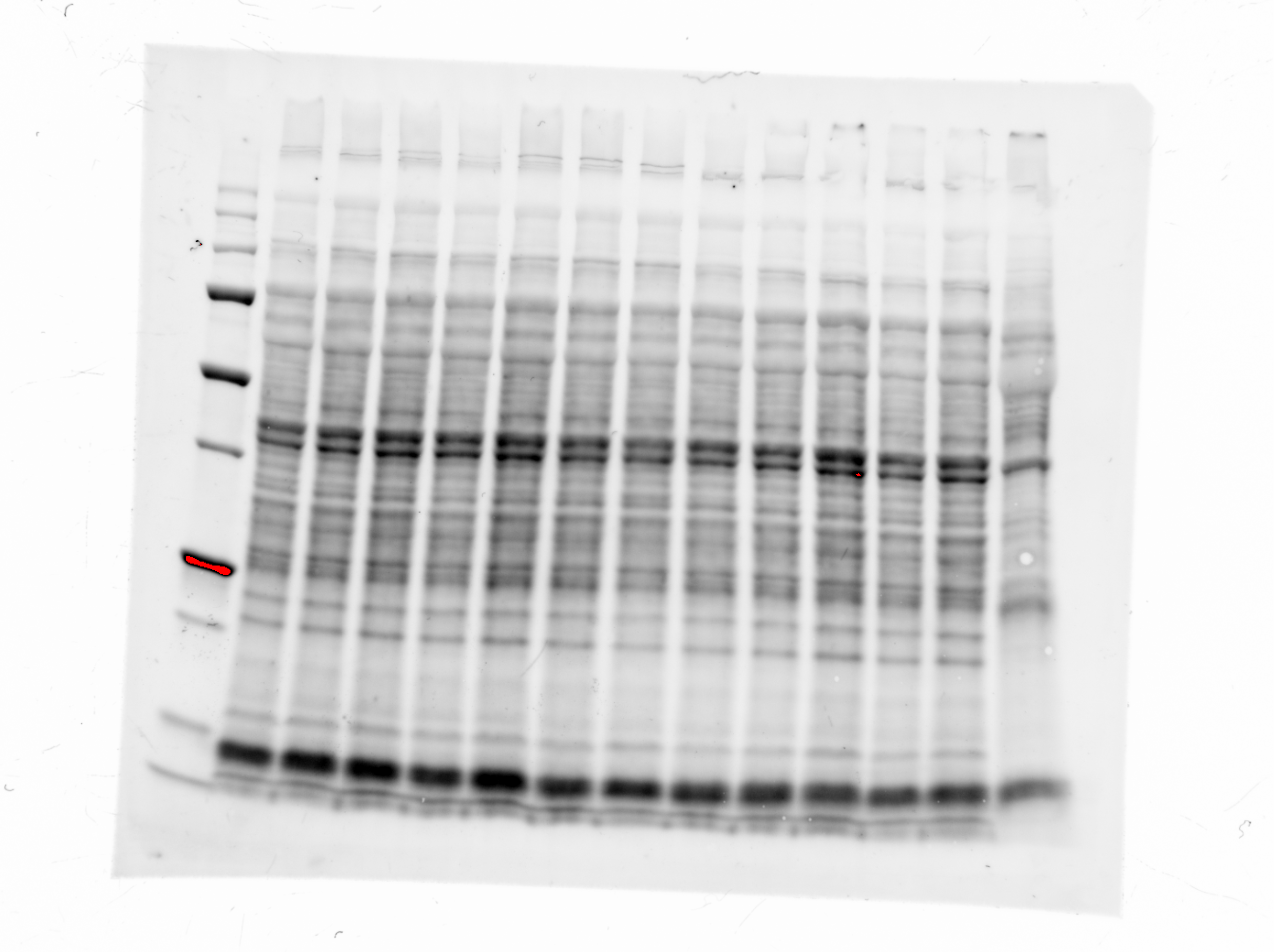

Supplement: Supplementary file 1 [file biology-11-00623-s001.zip › Figure S2.tif]
